# Anticoagulation in patients with atrial fibrillation and heart failure: Insights from the NCDR PINNACLE‐AF registry

**DOI:** 10.1002/clc.23142

**Published:** 2019-01-30

**Authors:** Johanna P. Contreras, Kimberly N. Hong, Javier Castillo, Lucas N. Marzec, Jonathan C. Hsu, Christopher P. Cannon, Song Yang, Thomas M. Maddox

**Affiliations:** ^1^ Mount Sinai School of Medicine, Division of Cardiology New York New York; ^2^ University of Colorado School of Medicine, Division of Cardiology Aurora Colorado; ^3^ University of California, Division of Cardiovascular Medicine San Diego California; ^4^ Harvard Medical School, Baim Institute for Clinical Research Boston Massachusetts

**Keywords:** anticoagulation, atrial fibrillation, congestive heart failure, heart failure with preserved ejection fraction, heart failure with reduced ejection fraction

## Abstract

**Background:**

In non‐valvular atrial fibrillation (NVAF) patients, congestive heart failure (CHF) confers an increased risk of stroke or systemic thromboembolism. This risk is present in both heart failure (HF) with reduced ejection fraction (HFrEF) and HF with preserved ejection fraction (HFpEF). It is unclear if clinicians account for both types of CHF in their NVAF anticoagulation practices. Accordingly, we characterized current outpatient anticoagulation trends in NVAF patients with HFpEF compared to patients with HFrEF.

**Methods:**

The outpatient NCDR PINNACLE‐AF registry was analyzed to identify patients with NVAF and CHF. The study population was subdivided into HFpEF (ie, LVEF ≥ 40%) and HFrEF (LVEF < 40%). Anticoagulation rates by CHF group were compared and stratified by CHA_2_DS_2_‐VASc score.

**Results:**

A total of 340 127 patients with NVAF and CHF were identified, of whom 248 136 (73.0%) were classified as HFpEF and 91 991 (27.0%) as HFrEF. Patients with HFpEF had higher mean CHA_2_DS_2_‐VASc scores and were more likely to be female, older, and have hypertension (*P* < 0.001). Unadjusted anticoagulation rates were significantly lower in patients with HFpEF compared to those with HFrEF (60.6% vs 64.2%, respectively). Lower rates of anticoagulation in the HFpEF group persisted after risk adjustment (RR: 0.93 [95% CI: 0.91, 0.94]). Stratification by CHA_2_DS_2_‐VASc score demonstrated that lower rates of anticoagulation in patients with HFpEF persisted until a score of ≥5.

**Conclusions:**

Patients with NVAF and HFpEF have significantly lower anticoagulation rates when compared to their HFrEF counterparts. These findings suggest a potential underappreciation of HFpEF as a risk factor in patients with NVAF.

**ABBREVIATIONS**
CAD: coronary artery disease
CI: confidence interval
CHF: congestive heart failure
DM: diabetes mellitus
HFrEF: heart failure with reduced ejection fraction
HFpEF: heart failure with preserved ejection fraction
HTN: hypertension
LVEF: left ventricular ejection fraction
NVAF: non‐valvular atrial fibrillation
RR: relative risk
TIA: transient ischemic attack

## 1 INTRODUCTION

Atrial fibrillation (AF) is the most common cardiac arrhythmia, with a reported 2% prevalence among all medicare beneficiaries <65 years old and 9% ≥65 years old, and an estimated 2‐10% annual stroke risk depending on individual risk factors.^1^, ^2^ Anticoagulation strategies for reducing the incidence of cerebral and systemic thromboembolism in the setting of non‐valvular AF (NVAF) have relied on risk score systems including the CHADS_2_ and the more current CHA_2_DS_2_‐VASc scores. The CHA_2_DS_2_‐VASc score, which has more discriminatory power in individuals characterized as low risk by CHADS_2_, was described and validated in 2009, and defines congestive heart failure (CHF) as a left ventricular ejection fraction (LVEF) ≤40%, or signs or symptoms of right or left heart failure (HF).^1^, ^3^, ^4^, ^5^ The American College of Cardiology/American Heart Association/Heart Rhythm Society (ACC/AHA/HRS) and European Society of Cardiology (ESC) all recommend using the CHA_2_DS_2_‐VASc score to risk stratify individuals with AF to inform anticoagulation decisions.^2^, ^5^ Specifically, the 2014 AHA/ACC/HRSA Guidelines for the Management of Patients with AF recommend (Class I) all individuals with a history of prior stroke or TIA, or CHA_2_DS_2_‐VASc score of ≥2 be considered for anticoagulation based on their bleeding risk, while the 2016 ESC Guidelines for the Management of AF recommends anticoagulation in females with a CHA_2_DS_2_‐VASc score ≥ 2 and males with a score ≥ 1.^1^, ^5^

Even though congestive HF has been validated as an independent risk factor for stroke regardless of ejection fraction, the clinical significance of HF with preserved ejection fraction (HFpEF) may be underappreciated. Approximately, 50% of all patients with HF have HFpEF, with cross‐sectional studies reporting rates between 40% to 71% depending on the EF cutoff (40%‐50%) used to define HFpEF, and longitudinal studies showing an increasing prevalence over time.^6^, ^7^, ^8^, ^9^, ^10^, ^11^

Practice patterns regarding the prescription of anticoagulation for patients with NVAF and HFpEF or HFrEF have not been well characterized and guideline adherence remains unknown. Prior studies in the HF population have reported varying rates of anticoagulation in those with NVAF, with one meta‐analysis that aggregated unadjusted data from 10 studies showing lower rates in the HFpEF compared to the HFrEF population, and another study, which showed after risk adjustment, equal rates of anticoagulation in inpatients with HFpEF or HFrEF (11, 17). The aim of this study was to characterize current outpatient practice patterns regarding the use of anticoagulation in a large cohort of outpatient patients with NVAF and CHF. By characterizing rates of oral anticoagulation in patients with HFpEF compared to HFrEF, we hope to identify gaps between practice patterns and anticoagulation guidelines in patients with AF and HF.

## 2 METHODS

### 2.1 Data collection

**Figure 1:**
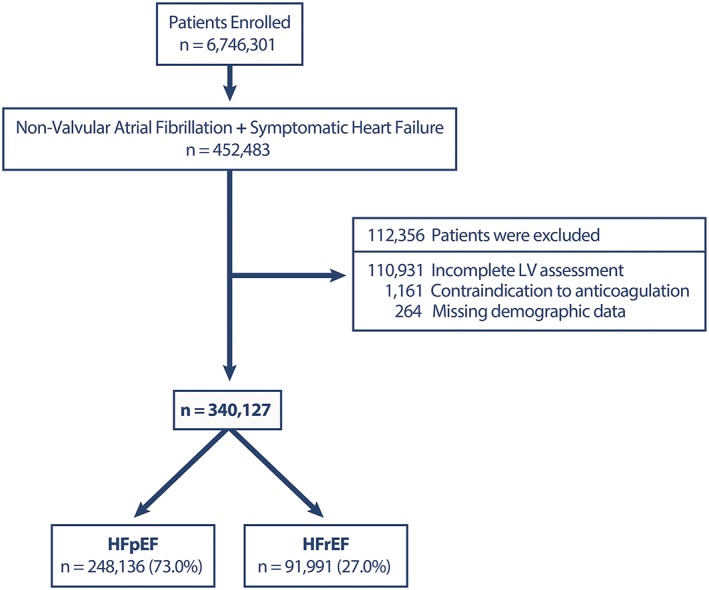
Flow chart of the study population. This flow chart shows how the study population was derived. HFpEF, heart failure with preserved ejection fraction; HFrEF, heart failure with reduced ejection fraction; LV left ventricle

We retrospectively analyzed a series of 6 746 301 consecutive patients who were prospectively entered into the American College of Cardiology (ACC), National Cardiovascular Data Registry (NCDR), and Practice Innovation and Clinical Excellence (PINNACLE) outpatient data registry between January 1 2008 to June 30 2016. The NCDR PINNACLE‐AF registry is a verified, peer‐reviewed data collection system that includes national adult outpatient visits. The initial study population included 452 483 patients with NVAF and CHF. Patients with incomplete left ventricular assessment (n = 110 931), any contraindication to therapeutic oral anticoagulation (n = 1161), or missing demographic data (n = 328) were excluded **(**Figure 1 **).** The final study population included 340 127 patients. Patient demographics and risk factors, baseline clinical characteristics, use of anticoagulation, and assessment of left ventricular function were obtained from the last outpatient visit during the January 1 2008 to June 30 2016 study period.

### 2.2 Definitions and study endpoints

The primary predictor variable was CHF group, with patients classified into two subgroups: HFpEF (LVEF ≥ 40%) and HFrEF (LVEF < 40%). The EF cutoff of 40% was chosen to differentiate between HFpEF and HFrEF because this was used as the objective definition of HF in the CHA_2_DS_2_‐VASc score. In addition, although epidemiological studies characterizing HFpEF have had variable definitions for HFpEF (>40% vs >50%), reduced EF was consistently defined as <40%.^6^, ^7^, ^8^, ^9^, ^10^ LVEF was obtained from electronic medical record data. CHF was defined as either symptoms or physical exam findings consistent with heart failure, a prior hospitalization with HF as a primary diagnosis, or an LVEF <40%. Patients without LVEF were excluded from the analysis as described above.

The CHA_2_DS_2_‐VASc score was calculated for each patient. One point was assigned for the presence of peripheral vascular disease, CHF, diabetes mellitus (DM), hypertension (HTN) and age 6 to ‐74 years. Two points were assigned if prior history of stroke/TIA or age ≥ 75 years old. As per the NCDR PINNACLE‐AF registry data standards, CHF was defined as symptoms of HF including dyspnea, orthopnea, rales, edema, S3, S4, ascites, or JVD, LVEF <40%, or exacerbation of CHF requiring hospitalization. In addition, vascular disease is defined as the presence of peripheral arterial disease, peripheral vascular disease, history of myocardial infarction, and prior interventional, or surgical revascularization.^12^

The main outcome parameter of the present study was rates of oral anticoagulation defined as warfarin, dabigatran, rivaroxaban, apixaban, or edoxaban. To better characterize how stroke risk and guideline recommendations impact rates of anticoagulation, secondary analyses included stratification by: (a) CHA_2_DS_2_‐VASc score, (b) CHA_2_DS_2_‐VASc ≤1 compared to CHA_2_DS_2_‐VASc ≥2, and (c) history of TIA or stroke compared to no prior TIA or stroke.^1^ To test the robustness of the results, sensitivity analysis excluding those on dual antiplatelets was done. In addition, to understand trends in anticoagulation over time, temporal analysis over the study period was conducted.

### 2.3 Statistical analysis

Continuous variables were reported as mean ± SD and compared using the Student's *t* test. Categorical variables were reported as percentages and compared using the *χ* ^2^ test or Fisher exact test. Given the large sample size, standardized differences between groups were calculated to report effective size to help with interpreting statistically significant differences. Standardized differences of <0.1 are generally considered to represent an insignificant effective size.^13^ Univariate, bivariate, and multivariable logistic regression analyses were performed to assess the effect of HF type (ie, HFrEF or HFpEF), CHA_2_DS_2_‐VASc, age, gender, body mass index, prior revascularization, coronary artery disease, prior myocardial infarction, cerebral thromboembolism, peripheral arterial disease, systemic thromboembolism, HTN, or DM on rates of anticoagulation. In the temporal analysis, HF type, year of last outpatient visit and respective interaction terms were included in the logistic regression model. The models accounted for clustering of patients by practice by using generalized estimating equations. To directly estimate risk ratios, Zou's method was used by specifying a Poisson distribution and including a robust variance estimate in the models. The risk‐adjusted anticoagulation rates were calculated using the ratio of observed: expected rates of anticoagulation. Summing the resultant coefficients from the function of independent variables included in the model estimated the individual risk probabilities of anticoagulation. The total expected rate of anticoagulation is derived by summing the individual risk probabilities for anticoagulation. The risk‐standardized anticoagulation rates were then calculated by multiplying the observed: expected rates by the average anticoagulation rate for the entire population. All reported *P*‐values are two‐sided. All data were analyzed using the statistical software package, SAS version 9.4 (SAS Institute, Cary, North Carolina). The Harvard Clinical Research Institute completed the analysis for this study.

## 3 RESULTS

### 3.1 Study population

A total of 340 127 patients with congestive HF and NVAF were included in the study. Of these, 248 136 (73.0%) and 91 991 (27.0%) were identified as patients with HFpEF and HFrEF, respectively. Table 1 shows all patient variables stratified by HF groups.

**Table 1:** Baseline characteristics by heart failure group

Variable	NVAF + CHF	HFpEF	HFrEF	Standardized difference	*P*‐value
Count	305 223	210 917 (69%)	94 306 (31%)	**—**	—
Age (years)	75.4 ± 11.6	76.0 ± 11.4	73.8 ± 11.8	**0.190**	<0.001
Male	59.6%	54.5%	73.4%	**−0.402**	<0.001
BMI (kg/m^2^)	29.2 ± 6.5	29.5 ± 6.6	28.6 ± 6.2	**0.131**	<0.001
Hypertension	294 912 (86.7%)	220 065 (88.7%)	74 847 (81.4%)	**0.206**	<0.001
Diabetes	106 840 (31.4%)	76 591 (30.9%)	30 249 (32.9%)	−0.043	<0.001
CKD	31 108 (9.1%)	21 758 (8.8%)	9350 (10.2%)	−0.048	<0.001
TIA/stroke	76 143 (22.4%)	56 914 (22.9%)	19 229 (20.9%)	0.049	<0.001
Systemic TE	4990 (1.5%)	3486 (1.4%)	1504 (1.6%)	−0.019	<0.001
Previous MI	84 940 (25.0%)	55 391 (22.3%)	29 549 (32.1%)	**−0.221**	<0.001
CAD	236 023 (69.4%)	165 753 (66.8%)	70 270 (76.4%)	**−0.214**	<0.001
PCI	70 887 (20.8%)	50 500 (20.4%)	20 387 (22.2%)	−0.044	<0.001
CABG	62 909 (18.5%)	41 434 (16.7%)	21 475 (23.3%)	**−0.167**	<0.001
NYHA class					<0.001
I	46 406 (13.6%)	40 818 (16.4%)	5588 (6.1%)	0.333	—
II	41 388 (12.2%)	30 379 (12.2%)	11 009 (12.0%)	0.008	—
III	17 060 (5.0%)	10 351(4.2%)	6709 (7.3%)	**−0.135**	—
IV	1973 (0.6%)	1076 (0.4%)	897 (1.0%)	−0.065	—
Dyspnea	208 697 (61.4%)	155 048 (62.5%)	53 649 (58.3%)	0.085	<0.001
Orthopnea	52 847 (15.5%)	38 005 (15.3%)	14 842 (16.1%)	−0.022	<0.001
Rales	23 398 (6.9%)	16 581 (6.7%)	6817 (7.4%)	−0.028	<0.001
Edema	151 711 (44.6%)	113 223 (45.6%)	38 488 (41.8%)	0.076	<0.001
S_3_Gallop	21 443 (6.3%)	13 936 (5.6%)	7507 (8.2%)	−0.101	<0.001
S_4_Gallop	29 107 (8.6%)	20 924 (8.4%)	8183 (8.9%)	−0.016	<0.001
Ascites	2754 (0.8%)	1799 (0.7%)	955 (1.0%)	−0.034	<0.001
JVD	22 522 (6.6%)	14 884 (6.0%)	7638 (8.3%)	−0.090	<0.001
LVEF (%)				3.439	<0.001
Mean ± SD	48.7 ± 15.1	56.1 ± 9.0	28.0 ± 7.3	—	—
Median (IQR)	52.0(38.0,60.0)	55.0(50.0,61.0)	30.0(23.0,35.0)	—	—
CHA_2_DS_2_‐VASc					
Mean ± SD	5.0 ± 1.6	5.0 ± 1.6	4.7 ± 1.6	**0.205**	<0.001
1	4568 (1.3%)	2737 (1.1%)	1831 (2.0%)	−0.072	—
2	19 323 (5.7%)	12 666 (5.1%)	6657 (7.2%)	−0.089	—
3	38 543 (11.3%)	25 910 (10.4%)	12 633 (13.7%)	**−0.101**	—
4	67 848 (19.9%)	47 659 (19.2%)	20 189 (21.9%)	−0.068	—
5	90 328 (26.6%)	67 248 (27.1%)	23 080 (25.1%)	0.046	—
6	60 369 (17.7%)	45 934 (18.5%)	14 435 (15.7%)	0.075	—
7	35 886 (10.6%)	27 522 (11.1%)	8364 (9.1%)	0.066	—
8	18 882 (5.6%)	14 785 (6.0%)	4097 (4.5%)	0.068	—
9	4380 (1.3%)	3675 (1.5%)	705 (0.8%)	0.068	—
DAPT	38 812 (11.4%)	25 554 (10.3%)	13 258 (14.4%)	**−0.125**	<0.001
Aspirin	200 510 (59.0%)	143 172 (57.7%)	57 338 (62.3%)	−0.095	<0.001
P2Y12	49 551 (14.6%)	32 841 (13.2%)	16 710 (18.2%)	−0.136	<0.001

BMI, body mass index; CABG, coronary artery bypass graft; CAD coronary artery disease; DAPT, dual antiplatelet therapy; IQR, interquartile range; JVD, jugular venous distention; LVEF, left ventricular ejection fraction; MI myocardial infarction; NYHA, New York Heart Association; PCI percutaneous coronary intervention; TIA transient ischemic attack; TE thromboembolism.

Patients with HFpEF were significantly older (76.0 ± 11.4 vs 73.8 ± 11.8 years), more likely to be female (45.5% vs 26.6%) and had significantly higher body mass indexes (BMI) (29.5 ± 6.6 vs 28.6 ± 6.2 kg/m^2^) than patients with HFrEF. CHA_2_DS_2_‐VASc score was significantly higher in patients with HFpEF. This was mainly driven by female gender, age, and hypertension (88.7% vs 81.4%, *P* < 0.001). In contrast, individuals with HFrEF were more likely to have higher rates of surrogates of ischemic heart disease including coronary artery disease, prior history of myocardial infarction, previous coronary artery bypass graft, or being on dual antiplatelet therapy. For the remaining risk factors, there was a statistically significant difference in frequency between both groups, but standardized differences were less than 0.1, suggesting a small effect.

**Figure 2:**
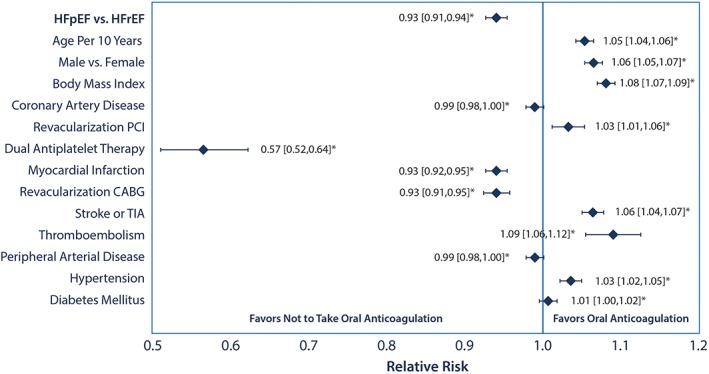
Relative risks for oral anticoagulation by baseline patient characteristics. Relative risks from the variables included in the multivariable model for predicting anticoagulation. CABG, coronary artery bypass graft; HFpEF, heart failure with preserved ejection fraction; HFrEF, heart failure with reduced ejection fraction, PCI percutaneous coronary intervention, TIA, transient ischemic attack

Overall unadjusted rates of anticoagulation were higher in the HFrEF group compared to the HFpEF group (64.2% vs 60.6%), even though mean CHA_2_DS_2_‐VASc score was higher in the HFpEF group (*P* < 0.001). After controlling for patient demographics, clinical variables, and CHA_2_DS_2_‐VASc components, individuals with HFpEF still remained significantly less likely to be anticoagulated than those with HFrEF (RR 0.93, 95% CI 0.91‐0.94). Age, BMI, HTN, previous cerebral or systemic thromboembolism, and prior percutaneous revascularization were all associated with increased rates of anticoagulation. Variables such as the use of dual antiplatelet therapy, prior myocardial infarction, and surgical revascularization were associated with lower rates of anticoagulation **(**Figure 2 **)**. These relationships persisted after exclusion of patients on dual antiplatelet therapy.

### 3.2 Secondary analyses

#### 3.2.1 CHA_2_DS_2_‐VASc

**Figure 3:**
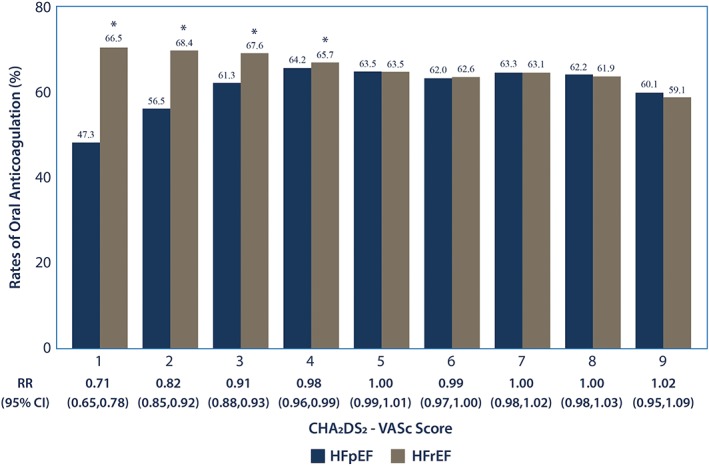
Risk adjusted rates of anticoagulation by heart failure group and CHA2DS2‐VASc score. HFpEF, heart failure with preserved ejection fraction; HFrEF heart failure with reduced ejection fraction

Analysis stratified by CHA_2_DS_2_‐VASc score showed lower observed rates of anticoagulation in HFpEF compared to HFrEF until CHA_2_DS_2_‐VASc score was 5 or greater. This relationship persisted even after risk adjustment, with the difference in anticoagulation rates being the greatest in the CHA_2_DS_2_‐VASc of 1 group and decreasing with each subsequent CHA_2_DS_2_‐VASc score until 5 **(**Figure 3). In the multivariable model that included only the CHA_2_DS_2_‐VASc components, increasing age, history of HTN, DM, stroke/TIA, or thromboembolism were all associated with increased use of anticoagulation (*P* < 0.001). Female gender and vascular disease were associated with lower rates (*P* < 0.001). See Table S1, Supporting Information for the CHA_2_DS_2_‐VASc component risk‐adjusted relative risks for anticoagulation.

Based on the AHA/ACC/HRSA recommendations that all individuals with a CHA_2_DS_2_‐VASc score of 2 or more, or history of stroke/TIA should be anticoagulated, separate analyses stratified by these groups were done. In the analysis stratified by CHA_2_DS_2_‐VASc score < 2 vs ≥2, the largest difference in unadjusted rates of anticoagulation between CHF groups was observed in individuals with a CHA_2_DS_2_‐VASc score of 1 (n = 4568), which in this study population were males with CHF as their sole thromboembolic risk factor. Individuals with HFpEF were less likely to be anticoagulated compared to those with HFrEF (46.1% vs 68.3%, *P* < 0.001). This relationship persisted after risk adjustment (RR 0.71, 95% CI 0.66‐0.76). Those on dual antiplatelet therapy were also less likely to be anticoagulated as expected (RR 0.52, 95% CI 0.35‐0.76). A larger BMI (RR 1.1, 95% CI 1.07‐1.13) and age (RR 1.18, 95% CI 1.14‐1.22) were both associated with increased rates of anticoagulation. Among patients with CHA_2_DS_2_‐VASc ≥2 (n = 335 559), individuals with HFpEF were similarly less likely to be anticoagulated compared to those with HFrEF (60.7% vs 64.1%, *P* < 0.001); however, the difference between anticoagulation rates was markedly smaller. This relationship persisted after risk adjustment (RR 0.93, 95% CI 0.92‐0.94). Risk factors associated with anticoagulation were not different from those found in the primary analysis.

#### 3.2.2 STROKE or TIA

In the sub‐analysis stratified by history of stroke/TIA, those with prior stroke/TIA (n = 76 143) were older (76.4 years vs 75.1 years) compared to those without prior stroke/TIA (n = 263 984). As expected the prior stroke/TIA cohort were also more likely to have cardiovascular risk factors including CAD, systemic embolism, prior revascularization, peripheral artery disease, HTN, DM, dyslipidemia, and chronic kidney disease, as well as higher rates of dual antiplatelets. Rates of anticoagulation in those with a prior history of stroke/TIA remained higher in the HFrEF group compared to the HFpEF group (65.3% vs 62.0%, RR 0.94, CI 0.92‐0.96, *P* < 0.001) even after controlling for risk factors. Risk factors associated with anticoagulation were similarly not different from those found in the primary analysis.

#### 3.2.3 Anticoagulation over time

**Figure 4:**
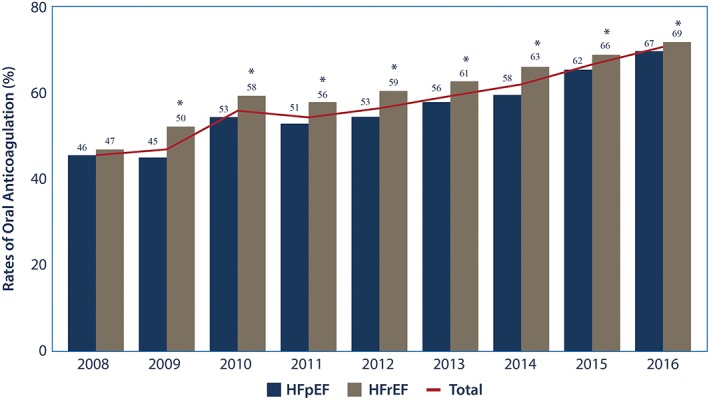
Observed rates of anticoagulation by heart failure group and year. This graphic shows the temporal trend in observed rates of anticoagulation in the congestive heart failure population (total and by heart failure group) with non‐valvular atrial fibrillation. Comparison between the observed rates of anticoagulation between the heart failure groups by year were made. *P < 0.05, HFpEF, heart failure with preserved ejection fraction; HFrEF heart failure with reduced ejection fraction

Unadjusted rates of anticoagulation have increased in both HFrEF and HFpEF groups over time, with higher rates of anticoagulation in the HFrEF group compared to the HFpEF group in all years except for 2008 (Figure 4). In the HFpEF group, anticoagulation rates increased by ~5.8% yearly (RR: 1.042, CI 1.031‐1.054, *P* < 0.001) and in the HFrEF group ~ 4.2% yearly (RR: 1.042, CI 1.031, 1.054).

## 4 DISCUSSION

Within our outpatient study population of individuals with NVAF and CHF, 69% of these patients had HFpEF. Although this falls within the upper range of what has been previously reported,^6^, ^7^, ^8^, ^9^, ^10^ the outpatient study cohort used was contemporary and is consistent with prior studies suggesting that HFpEF is a growing clinical entity. Importantly, our study showed that although HFpEF patients with NVAF had higher average CHA_2_DS_2_‐VASc scores, their overall rates of anticoagulation compared to their HFrEF counterparts were lower. Thus, even though data from observational studies and clinical trials show comparable rates of stroke/TIA within the reduced and preserved ejection fractions HF groups with AF,^14^, ^15^, ^16^ there remains a discrepancy in rates of anticoagulation between these groups in the outpatient. Importantly, even though HFpEF patients are less likely to be anticoagulated than HFrEF patients, rates of anticoagulation in both groups has increased over time.

Prior studies evaluating rates of anticoagulation in HF patients with AF have shown variable anticoagulation rates depending on the study population evaluated and whether or not the data were risk adjusted.^6^, ^14^, ^15^ In one observational study by Steinberg et al. which used inpatient registry data, lower unadjusted rate of anticoagulation in the HFpEF group compared to the HFrEF group resolved after risk adjustment.^6^ In the Sartipy et al study which used inpatient and outpatient data unadjusted rates of anticoagulation in patients with HFrEF were higher compared to those with HFpEF.^15^ Interestingly, this study found in multivariable analysis that anticoagulant use in patients with HFpEF was more likely to be associated with NVAF than in patients with HFrEF.^15^ This suggests that the HFrEF population may be more likely to have additional indications for anticoagulation compared to the HFpEF group, and may be why differences in anticoagulation rates between these two groups decrease after risk adjustment.

Our study provides insight into current outpatient anticoagulation patterns in patients with HF and AF. Although it is unclear why patients with HFpEF are less likely to be anticoagulated than those with HFrEF, this difference suggests that opportunities for improving rates of anticoagulation in this patient population exist. Central to the pathogenesis of HFpEF is the systemic inflammation resulting from the comorbid conditions including obesity, DM, HTN, and renal failure associated with it.^17^ Because of its heterogeneity, it is possible that patients with HFpEF are being underdiagnosed and therefore undertreated. Suggestive of this, was how the difference in anticoagulation prescription between HF groups narrowed as CHA_2_DS_2_‐VASc score increased. Specifically, in the CHA_2_DS_2_‐VASc = 1 group the difference in the risk‐adjusted rates of anticoagulation was nearly 20%, but decreased to 1.5% by CHA_2_DS_2_‐VASc = 4 before becoming and staying insignificant by CHA_2_DS_2_‐VASc ≥5. This discrepancy in the CHA_2_DS_2_‐VASc = 1 may be because a diagnosis of HFpEF may be more nuanced than HFrEF, and the accurate categorization of HFpEF may be more difficult for unexperienced clinicians. Contributing to these differences in anticoagulation rates may also be that the major guideline recommendations are less stringent in this lower risk group. Specifically, the ACC/AHA/HRS only recommends consideration of anticoagulation, aspirin or no antithrombotic therapy in individuals with a CHA_2_DS_2_‐VASc score of 1, and the ESC only recommends anticoagulation in males with CHA_2_DS_2_‐VASc score of 1. Regardless, a greater awareness of the stroke risk that HFpEF confers in NVAF may be needed.

In the analysis stratified by AHA/ACC/HRSA indications for anticoagulation in AF, specifically CHA_2_DS_2_‐VASc score 2 or greater, or prior stroke/TIA, less than two‐third of individuals who met a Class I indication for anticoagulation were being anticoagulated. Future studies should be aimed at further discriminating stroke/TIA risk in individuals categorized as low‐risk based on our current risk scores, so that standardized recommendations for anticoagulation can be made.

Our study has several limitations. This study involved an outpatient data registry that relies on manual entry and standardized algorithms for extracting data from the electronic medical records, so data completion was not 100%. Thus, the results only characterize outpatient patterns of anticoagulation prescription and are not generalizable to the entire population. In addition, HF classification relied on both quantitative and qualitative assessments of ejection fraction, and misclassification may have occurred. In addition, because this is not a prospective dataset being collected for the hypothesis being evaluated, there are data constraints including an inability to assess patient preferences, as well as other indications for anticoagulation, such as deep venous thromboembolism in our dataset. Resultantly, it was not possible to discriminate between and exclude individuals who had a contraindication to anticoagulation or an indication to anticoagulation other than AF in this dataset. Instead, as is convention with analyses using the PINNACLE dataset, we relied on a data field indicating medical contraindication to anticoagulation to exclude individuals from analysis. Longitudinal data was also not present, and instead only a snapshot of anticoagulation rates could be provided, and no outcomes data including rates of stroke/TIA, or medication adherence was available.

## 5 CONCLUSIONS

The majority of outpatients with NVAF and CHF have HFpEF, and even though these individuals have higher CHA_2_DS_2_‐VASc scores, they are significantly less likely to be prescribed oral anticoagulation compared to HFrEF patients. The discrepancy in anticoagulation rate is most prevalent in the lower risk groups, and may be secondary to differences in guideline recommendations and evidence for anticoagulating individuals when bleeding is a risk. Future studies are needed to clarify why differences exist in anticoagulation by HF type; however, our data calls for educating and alerting clinicians of this important risk factor for stroke prevention.

## 6 CLINICAL PERSPECTIVES

Congestive HF has been validated as an independent risk factor for stroke regardless of ejection fraction; however, the clinical significance of HF with preserved EF may be underappreciated. The majority of outpatients with NVAF and congestive HF have preserved ejection fraction. Even though these individuals have higher CHA2DS2‐VASc scores, they are significantly less likely to be prescribed oral anticoagulation compared to outpatients with HF with reduced EF which suggests an opportunity for educating and alerting clinicians of this important risk factor for stroke prevention.

## 7 TRANSLATIONAL OUTLOOK

Future studies are needed to clarify why differences exist in anticoagulation by HF type, and in particular why individuals with AF and HF with preserved EF are less likely to anticoagulated than their reduced EF counterparts.

## CONFLICTS OF INTEREST

The authors declare no potential conflict of interests.

## Supporting information


**TABLE S1** Adjusted model of OAC by HFpEF vs HFrEF using CHA_2_DS_2_‐VASc components
