# Supplementary material for: Anticoagulation in patients with atrial fibrillation and heart failure: Insights from the NCDR PINNACLE‐AF registry
Source: Clin Cardiol. 2019 Jan 30;42(3):339–45. doi: 10.1002/clc.23142 (PMC6712307; doi:10.1002/clc.23142)
Supplement: Supplementary file 1 — TABLE S1 Adjusted model of OAC by HFpEF vs HFrEF using CHA2DS2‐VASc components [file CLC-42-339-s001.docx]

Supplemental Online Table. Adjusted Model of OAC by HFpEF vs. HFrEF Using CHA_2_DS_2_-VASc Components

HFpEF heart failure with preserved ejection fraction, HFrEF heart failure with reduced ejection fraction, TIA transient ischemic attack
